# Reliability of transcardiopulmonary thermodilution cardiac output measurement in experimental aortic valve insufficiency

**DOI:** 10.1371/journal.pone.0186481

**Published:** 2017-10-19

**Authors:** Martin Petzoldt, Constantin J. Trepte, Jan Ridder, Stefan Maisch, Philipp Klapsing, Jan F. Kersten, Hans Peter Richter, Jens C. Kubitz, Daniel A. Reuter, Matthias S. Goepfert

**Affiliations:** 1 Department of Anesthesiology, Center of Anesthesiology and Intensive Care Medicine, University Medical Center Hamburg-Eppendorf, Hamburg, Germany; 2 Department of Medical Biometry and Epidemiology, University Medical Center Hamburg-Eppendorf, Hamburg, Germany; Kurume University School of Medicine, JAPAN

## Abstract

**Background:**

Monitoring cardiac output (CO) is important to optimize hemodynamic function in critically ill patients. The prevalence of aortic valve insufficiency (AI) is rising in the aging population. However, reliability of CO monitoring techniques in AI is unknown. The aim of this study was to investigate the impact of AI on accuracy, precision, and trending ability of transcardiopulmonary thermodilution-derived CO_TCPTD_ in comparison with pulmonary artery catheter thermodilution CO_PAC_.

**Methods:**

Sixteen anesthetized domestic pigs were subjected to serial simultaneous measurements of CO_PAC_ and CO_TCPTD_. In a novel experimental model, AI was induced by retraction of an expanded Dormia basket in the aortic valve annulus. The Dormia basket was delivered via a Judkins catheter guided by substernal epicardial echocardiography. High (HPC), moderate (MPC) and low cardiac preload conditions (LPC) were induced by fluid unloading (20 ml kg^-1^ blood withdrawal) and loading (subsequent retransfusion of the shed blood and additional infusion of 20 ml kg^-1^ hydroxyethyl starch). Within each preload condition CO was measured before and after the onset of AI. For statistical analysis, we used a mixed model analysis of variance, Bland-Altman analysis, the percentage error and concordance analysis.

**Results:**

Experimental AI had a mean regurgitant volume of 33.6 ± 12.0 ml and regurgitant fraction of 42.9 ± 12.6%. The percentage error between CO_TCPTD_ and CO_PAC_ during competent valve function and after induction of substantial AI was: HPC 17.7% vs. 20.0%, MPC 20.5% vs. 26.1%, LPC 26.5% vs. 28.1% (pooled data: 22.5% vs. 24.1%). The ability to trend CO-changes induced by fluid loading and unloading did not differ between baseline and AI (concordance rate 95.8% during both conditions).

**Conclusion:**

Despite substantial AI, transcardiopulmonary thermodilution reliably measured CO under various cardiac preload conditions with a good ability to trend CO changes in a porcine model. CO_TCPTD_ and CO_PAC_ were interchangeable in substantial AI.

## Introduction

In critically ill patients monitoring cardiac output (CO) can be the keystone in hemodynamic assessment and therapy in the operating theater or on the intensive care unit [1–3]. The accuracy, precision of agreement, and trending ability of measurement technologies are essential prerequisites. The prevalence of valvular heart diseases is rising rapidly in industrialized countries as a result of demographic changes [4, 5]. However, remarkably little is known about the reliability of CO monitoring devices in patients with valvular heart disease, particularly in those with aortic valve insufficiency (AI) [6–10].

Thermodilution techniques, either transcardiopulmonary thermodilution or pulmonary artery thermodilution, are established methods to measure CO at the bedside [11]. However, a methodological weakness is that the thermal indicator (i.e. ice-cold normal saline injection) may escape from the circulation due to conductive rewarming by the surrounding tissues [12, 13]. Thermal loss by conductive rewarming is considered to be promoted by the cyclic movement of the indicator in valvular regurgitation (prolonged travel of the indicator with increased escape to surrounding tissues) [12, 14].

Pulmonary artery thermodilution (CO_PAC_), which is regarded as the clinical gold standard method, is generally considered to be unreliable in the presence of significant tricuspid insufficiency [12, 15, 16], but due to the right cardiac measuring site it is not directly affected by aortic insufficiency.

For transcardiopulmonary thermodilution (CO_TCPTD_), the thermal indicator further travels through the lungs and left cardiac chambers to finally reach a thermistor downstream in a proximal artery. It is still unclear if (in analogy to right cardiac thermodilution via pulmonary artery catheter in tricuspid insufficiency) CO_TCPTD_ is substantially confounded by left cardiac regurgitation in AI, and further, if this relation is affected by cardiac preload conditions.

Therefore, the aim of this study was to investigate the impact of AI on accuracy, precision of agreement, and trending ability of CO_TCPTD_ in comparison with CO_PAC_ under varying cardiac preload conditions.

## Materials and methods

The study protocol was approved by the local governmental Animal Care and Use Committee of Hamburg as part of a larger experimental project (approval number 87/08). Sixteen healthy domestic pigs were studied. The care and handling of the animals were in strict accordance with the European Convention for the Protection of Vertebrate Animals used for Experimental and Other Scientific Purposes (Strasbourg, France). All efforts were made to minimize suffering. Experiments were carried out according to the ARRIVE guidelines [17].

### Anesthesia and surgical preparation

The animals were fasted overnight. Premedication was achieved by intramuscular injection of 250 mg s-ketamine, 360 mg azaperone and 15 mg midazolam. Subsequently all animals received a tracheostomy. Anesthesia was maintained with 1.5–2.0 end-tidal vol% of sevoflurane and intravenous infusion of 0.4 mg h^-1^ fentanyl. To facilitate surgical preparation 4 mg h^-1^ pancuronium bromide was administered. Animals were mechanically ventilated in a volume autoflow mode (Zeus^TM^, Draeger Medical, Luebeck, Germany) via an endotracheal tube (8.0 mm inner diameter). Mechanical ventilation was set to a tidal volume of 8 ml kg^-1^, a positive end-expiratory pressure of 5 cmH_2_O, an inspiration to expiration ratio of 1:2, and a fraction of inspired oxygen of 0.4. Respiratory rate was adjusted to maintain end-tidal carbon dioxide partial pressure between 35 and 40 mmHg. Routine monitoring included 5-lead electrocardiogram, pulse oximetry and side-stream capnography. 13 ml kg^-1^ h^-1^ saline infusion was given to maintain periprocedural hydration. Furthermore, during instrumentation and surgical preparation the animals received an unrestricted amount of hydroxyethyl starch to maintain stable hemodynamic conditions without vasopressor application, and a stroke volume variation ≤12%. In each animal 1 g cefuroxime, 250 mg prednisolone, 5,000 units of unfractionated heparin and 500 mg acetylsalicylic acid were administered.

For instrumentation and surgical preparation animals were placed in a supine position. A 7-French central venous catheter (Certofix^TM^ Trio V 730, B. Braun Melsungen AG, Melsungen, Germany) was inserted into the left-sided internal jugular vein and central venous pressure (CVP) was continuously measured. Aortic pressure was assessed using a microtip catheter (SPC 350, Millar Instruments, Houston, TX) positioned in the aortic arch via an 5-Fr introducer sheath (Intradyn^TM^, Venous Hemostasis Introducer, B. Braun) placed in the left internal carotid artery. In each animal a pulmonary artery catheter (Intrathermodin^TM^, 4-lumen, 7-Fr, 110 cm; Intraspecial Catheters GmbH, Rehlingen-Siersburg, Germany) was inserted via an 8-Fr introducer sheath (Intradyn^TM^, B. Braun) placed in the left external jugular vein. Obtained values were displayed on the vital signs monitor (Infinity Delta; Draeger AG, Luebeck, Germany). A thermistor-tipped cardiac output catheter (5-Fr, Pulsiocath^TM^, Pulsion Medical Systems SE, Feldkirchen, Germany) was inserted via the right iliac artery and connected to the corresponding hemodynamic monitor (PiCCO^TM^, version 7.1, Pulsion Medical Systems) to measure CO_TCPTD_. Body temperature was measured via this arterial catheter and kept constant.

After sternotomy and opening the pericardium the animals received aortic ultrasonic flow probes, which were used for another study protocol [18] but could not be applied for the present investigation due to interference with epicardial echocardiography. An Omniplane III TEE probe was introduced directly from the outside and positioned beneath the cardiac apex to enable substernal epicardial echocardiography (SONOS 5500^TM^, Philips Medical Systems, Andover, USA). Subsequently, the pericardium was reconstructed with a patch, the sternum was reclosed with wire cerclages, and the thorax was closed prior to study measurements.

**Fig 1:**
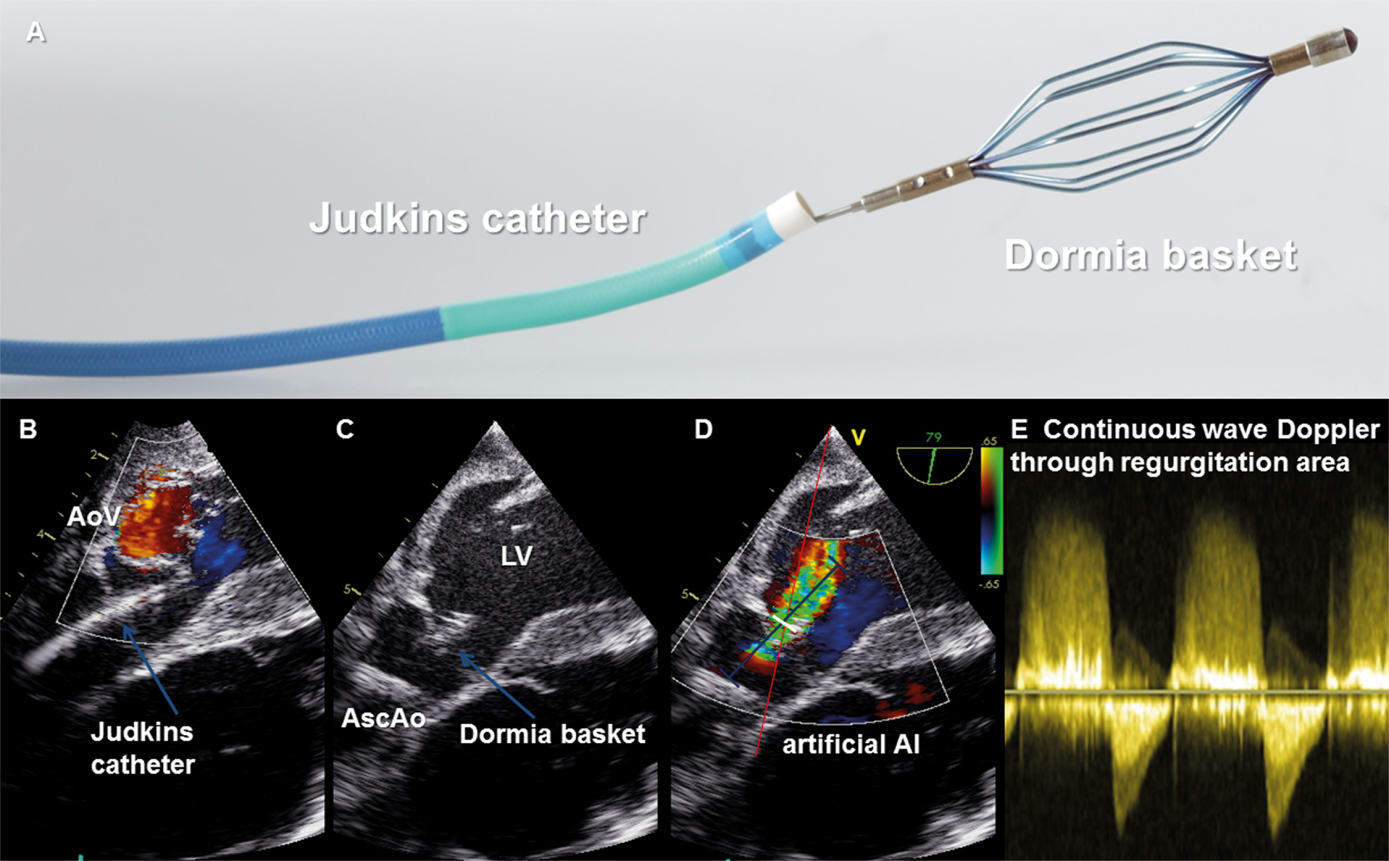
Porcine model for experimental aortic valve insufficiency. (A) A Judkins catheter was used as a guiding catheter to deliver a Dormia basket, (B) The Judkins catheter was introduced via an introducer sheath in the carotid artery and advanced through the brachiocephalic trunk into the ascending aorta (AscAo), (C) A compressed Dormia basket was delivered via the Judkins catheter through the aortic valve (AoV) in the left ventricle (LV). Subsequently the expanded Dormia basket was retracted in the aortic valve annulus, (D) Targeted tip position for the Dormia basket to induce substantial aortic valve regurgitation verified by epicardial echocardiography (E).

A Judkins catheter (Vista Brite^TM^ Tip Guiding Catheter JR 4 SH 7F, Cordis, Miami; FL) was inserted via another 10-Fr introducer sheath (right internal carotid artery) and was advanced through the brachiocephalic trunk into the ascending aorta. This Judkins catheter was used to deliver a compressed Dormia basket (EPflex^TM^ Feinwerktechnik GmbH, Dettingen, Germany) with a diameter of 5 to 8 mm (wedged) through the aortic valve in the left ventricle (Fig 1). Subsequently the expanded Dormia basket was retracted in the aortic valve annulus to induce substantial AI guided by epicardial echocardiography.

### Measurements and experimental protocol

A high flow hemodialysis catheter (Certofix^TM^ Trio HF S 1220, B. Braun) was placed in the right femoral vein for rapid fluid unloading or loading. Three cardiac preload conditions were induced at predefined measuring times:

1. Moderate cardiac preload conditions (MPC): baseline values after instrumentation and surgical preparation.
2. Low cardiac preload conditions (LPC): induced via fluid unloading by 20 ml kg^-1^ blood withdrawal.
3. High cardiac preload conditions (HPC): induced via subsequent fluid loading by retransfusion of the shed blood and additional infusion of 20 ml kg^-1^ hydroxyethyl starch.

AI was induced within each preload condition and forward stroke volume, total stroke volume, regurgitant volume, regurgitation fraction and pressure half time were assessed as the mean of three echocardiographic measurements prior to study measurements. Echocardiography parameters were not assessed simultaneously with thermodilution parameters.

The combination of three preload and two valve conditions (competent valve and AI) gave a total of six predefined measuring times. We waited for at least 5 minutes after each loading or unloading, on- or offset of AI to allow hemodynamic equilibration prior to study measurements. Within each measuring time mean aortic pressure (MAP), aortic pulse pressure, CVP, heart rate (HR), and mean pulmonary arterial pressure (mPAP) were measured along with CO_PAC_ and CO_TCPTD_. For simultaneous assessment of CO_TCPTD_ and CO_PAC_ three sequential 10-ml cold (8°C) sodium chloride injections were randomly delivered throughout the respiratory cycle via the proximal port of the pulmonary artery catheter.

After completion of the experimental protocol the animals were killed by fast injection of 40 mmol potassium chloride during deep anesthesia.

### Statistical analysis

Data were checked for normal distribution. Continuous data are presented as mean ± standard deviation (SD). Categorical data are presented as frequencies (n) or percentage values (%).

Analyses of variance of hemodynamic data were used. To eliminate the problem of correlated within-subject errors during repeated measures in single animals, differences between hemodynamic baseline parameters at six predefined measuring times were analyzed within mixed models with the individual animals as random effects, and the animal weight as a fixed effect. Thus the models are adjusted for weight.

The Bland-Altman method accounting for repeated measurements within single subjects [19] was used to evaluate agreement between CO_TCPTD_ and CO_PAC_ (reference technology) expressed as the mean of the differences (bias) with 95% limits of agreement (calculated as bias ± 1.96 x SD). The percentage error was calculated as described by Critchley and Critchley [20]. A percentage error of up to 30% has been suggested to define clinically acceptable agreement between study and reference technology in CO method comparison studies [20].

To assess the trending capability of CO_TCPTD_ in conditions with and without AI we created four-quadrant plots including both preload interventions (fluid loading and unloading). Concordance analysis was performed as described previously respecting a predefined exclusion zone of 0.5 l min^-1^ [21–23]. A concordance rate >92% has been proposed to indicate good trending ability [21].

Statistical analysis was performed using SPSS for Windows Release 22.0.0 (IBM SPSS Inc, Chicago, IL, USA), SigmaPlot 12.0 (Systat Software Inc, San Jose, CA, USA) and the statistical analysis software R 3.2.3. [24]. Statistical significance was accepted at a level of 0.05.

## Results

The hemodynamics of sixteen healthy domestic pigs weighing 50.4 ± 7.2 kg were analyzed. After the onset of AI in hypovolemia one of these pig hemodynamically deteriorated (no. 7) and another one died (no. 3). In both animals subsequent data sets (i.e. hemodynamic measurements during AI in LPC and in HPC) could not be gathered.

We were able to induce substantial AI in our porcine model (moderate to severe AI in 84.1% of the cases) with a mean regurgitant volume of 33.6 ± 12.0 ml corresponding to a regurgitant fraction of 42.9 ± 12.6%. AI was fully reversible after removal of the Dormia basket. Echocardiographic findings are summarized in Table 1.

**Table 1:** Grading of experimental aortic valve insufficiency.

Echocardiographic parameter	mean ± SD n = 44
Degree of aortic valve insufficiency (mild/moderate/severe) [n]	7/23/14
Moderate to severe aortic valve insufficiency [%]	84.1
Total stroke volume [ml]	81.7 ± 30.1
Forward stroke volume [ml]	48.0 ± 26.8
Regurgitant volume [ml]	33.6 ± 12.0
Regurgitation fraction [%]	42.9 ± 12.6
Pressure half time [ms] ^a^	183.2 ± 71.8

Measurement were performed during experimental aortic valve insufficiency at 3 predefined measuring times in 16 animals (n = 44)

^a^: n = 43 values due to 1 missing value, values are presented as mean ± standard deviation (SD); categorical data are presented as frequencies [n] or percentage values [%]; the degree of aortic valve insufficiency was defined as recommended by the American College of Cardiology/American Heart Association [4]: severe: Regurgitation fraction (RF) ≥ 50%, moderate: RF: 30–49%, mild: RF <30%

### Hemodynamic baseline parameters

Our variance components model (mixed model) showed (Table 2):

- Variations due to experimental induction of AI:

CO_PAC_, CO_TCPTD_ and MAP decreased after induction of AI. Aortic pulse pressure significantly increased after the onset of AI. HR and mPAP were not relevantly affected by experimental induction of AI.

- Variations due to cardiac preload changes:

LPC was associated with a significantly lower CO_PAC_, CO_TCPTD_, MAP, mPAP and CVP and higher HR compared with HPC. Aortic pulse pressure was not relevantly affected by preload changes.

**Table 2:** Hemodynamic changes related to induction of aortic insufficiency and cardiac preload changes (mixed model).

Variable	Preload conditions	Aortic insufficiency (AI)	Baseline	Difference AI vs. baseline^a^	P-Value^a^	Difference low vs. high	P-value^b^
**CO_PAC_** [l min^-1^]	High (HPC)	4.28 ± 1.36	4.64 ± 1.40	-0.36 ± 0.47	p = 0.005	-1.4 ± 1.2	p<0.001
	Medium (MPC)	4.03 ± 0.70	4.44 ± 0.64	-0.41 ± 0.26	p<0.001		
	Low (LPC)	2.89 ± 0.76	3.20 ± 0.89	-0.31 ± 0.19	p = 0.009		
**CO_TCPTD_** [l min^-1^]	High (HPC)	4.95 ± 1.48	5.42 ± 1.55	-0.47 ± 0.48	p = 0.004	-1.3 ± 1.4	p<0.001
	Medium (MPC)	4.75 ± 0.80	5.12 ± 0.73	-0.37 ± 0.30	p = 0.002		
	Low (LPC)	3.71 ± 1.09	4.00 ± 0.99	-0.29 ± 0.39	p = 0.171		
**MAP** [mmHg]	High (HPC)	62.5 ± 14.2	69.4 ± 14.8	-7.0 ± 4.5	p<0.001	-10.9 ± 14.0	p = 0.005
	Medium (MPC)	70.5 ± 9.3	76.0 ± 9.6	-5.5 ± 5.2	p<0.001		
	Low (LPC)	51.6 ± 11.2	53.8 ± 8.4	-2.2 ± 3.8	p = 0.071		
**PP** [mmHg]	High (HPC)	54.2 ± 10.7	48.3 ± 8.5	5.9 ± 8.2	p = 0.009	-0.6 ± 18.1	p = 0.886
	Medium (MPC)	61.7 ± 11.0	47.1 ± 8.0	14.6 ± 6.5	p<0.001		
	Low (LPC)	53.6 ± 17.1	43.4 ± 10.5	10.3 ± 8.8	p<0.001		
**CVP** [mmHg]	High (HPC)	16.0 ± 3.4	14.8 ± 4.6	1.2 ± 3.1	p<0.001	-9.3 ± 2.1	p<0.001
	Medium (MPC)	10.3 ± 3.1	10.8 ± 3.4	-0.5 ± 0.6	p = 0.248		
	Low (LPC)	6.8 ± 2.4	6.7 ± 2.4	0.1 ± 0.4	p = 0.013		
**HR** [min^-1^]	High (HPC)	93.7 ± 9.7	95.7 ± 10.7	-1.9 ± 3.8	p = 0.254	25.3 ± 17.0	p<0.001
	Medium (MPC)	97.8 ± 13.6	98.2 ± 12.7	-0.5 ± 5.3	p = 0.771		
	Low (LPC)	119.1 ± 20.5	119.9 ± 18.5	-0.9 ± 3.7	p = 0.972		
**mPAP** [mmHg]	High (HPC)	30.7 ± 5.2	29.9 ± 7.6	0.8 ± 3.4	p = 0.076	-7.4 ± 3.8	p<0.001
	Medium (MPC)	27.2 ± 6.2	27.1 ± 6.4	0.1 ± 1.3	p = 0.735		
	Low (LPC)	23.3 ± 5.4	23.6 ± 5.7	-0.2 ± 3.2	p = 0.073		

CO_PAC_: pulmonary artery catheter derived cardiac output; CO_TCPTD_: transcardiopulmonary thermodilution derived cardiac output; MAP: mean aortic pressure; PP: aortic pulse pressure; CVP: central venous pressure; HR: heart rate; mPAP: mean pulmonary arterial pressure

^a^: Difference between measurement values assessed during aortic valve insufficiency (AI) compared with baseline conditions

^b^: Difference between values assessed during low preload conditions (LPC) compared with high preload conditions (HPC) in conditions of AI

Values are presented as mean ± standard deviation (SD); differences between measurement times (change in valve and/or preload conditions) were tested within mixed models with animals as random effects, adjusted for animal weight; statistical significance was accepted at a level of p = 0.05

### Agreement between CO_TCPTD_ and CO_PAC_

**Fig 2:**
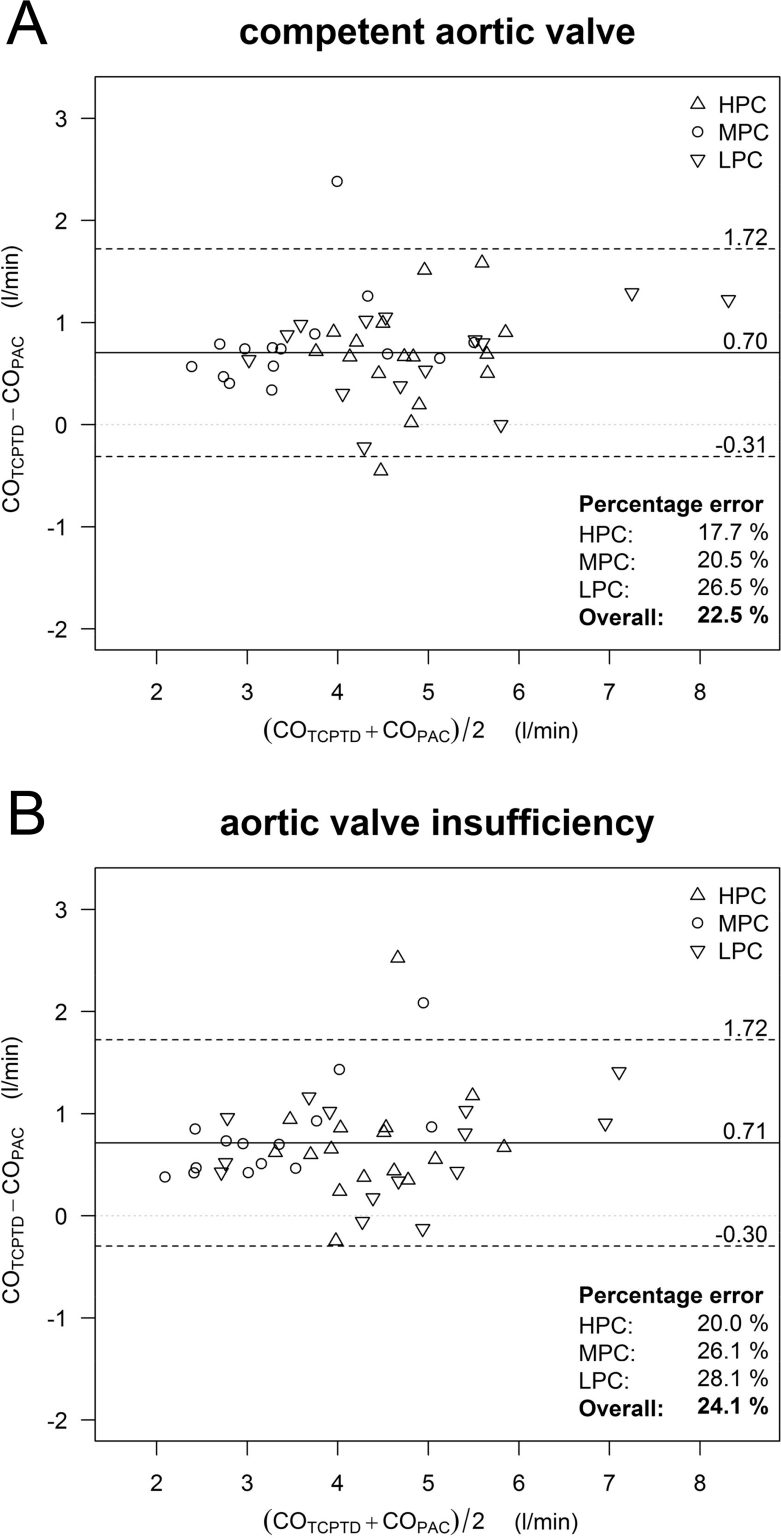
Bland-Altman-plots. Bland-Altman-plots accounting for repeated measurements within single subjects illustrate agreement between cardiac output (CO) derived from transcardiopulmonary thermodilution (CO_TCPTD_) and pulmonary artery thermodilution (CO_PAC,_ reference method) (A) under baseline conditions (above, n = 45 data pairs) and (B) immediately after induction of aortic valve insufficiency (below, n = 44 data pairs). Data were sampled under various preload conditions (high [HPC]; moderate [MPC] and low [LPC] preload conditions) and analyzed separately or as pooled data. The continuous horizontal line shows the mean bias between both methods, while the dashed horizontal lines show the upper and lower 95% limits of agreement (bias ± 1.96 × standard deviation).

Bland-Altman-plots illustrate the agreement between the CO_TCPTD_ and CO_PAC_ before and after the onset of AI (Fig 2). Irrespective of the preload or valve conditions a fixed bias between CO_TCPTD_ and CO_PAC_ (0.73 l min^-1^ without and 0.71 l min^-1^ with AI) was found. The percentage errors between CO_TCPTD_ and CO_PAC_ during baseline conditions (competent valve) and after induction of substantial AI were: HPC 17.7% vs. 20.0%, MPC 20.5% vs. 26.1%, LPC 26.5% vs. 28.1%, indicating clinically acceptable agreement between methods during all preload and valve conditions [20].

### Trending ability of CO_TCPTD_

**Fig 3:**
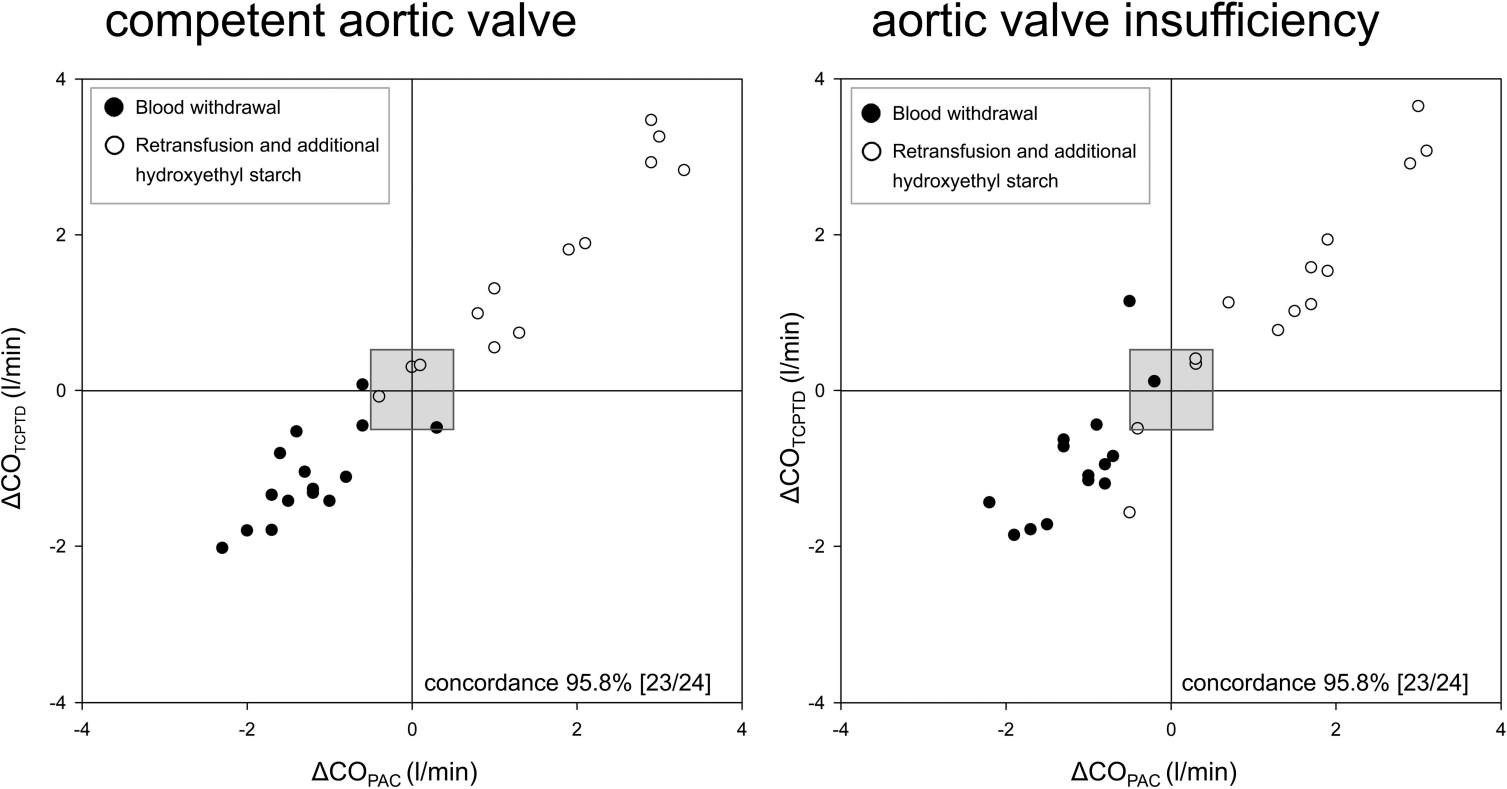
Four-quadrant plots. Trending ability of cardiac output (CO) derived from transcardiopulmonary thermodilution (CO_TCPTP_) compaired with CO_PAC_ (reference method) illustrated by four-quadrant plots. The ability to trend CO changes induced by preload changes was assessed during baseline conditions (left: competent aortic valve) and after induction of aortic valve insufficiency (right). Changes in cardiac preload were induced by fluid unloading (black dots: withdrawal of 20 ml kg^-1^ blood) and subsequent fluid loading (white dots: retransfusion of the shed blood and additional infusion of 20 ml kg^-1^ hydroxyethyl starch). The concordance analysis gives a concordance rate of 95.8% during both conditions, baseline and aortic valve insufficiency. An exclusion zone of 0.5 l min^-1^ (grey area in the center) was applied.

The ability of CO_TCPTD_ to trend CO changes induced by preload changes is illustrated in four-quadrant plots (Fig 3). The concordance rate was 95.8% under baseline conditions (competent valve) as well as in conditions with substantial AI. This indicates good trending ability, according to Critchley et al. [21], for CO_TCPTD_ in conditions with and without AI.

## Discussion

This experimental study systematically compared CO_TCPTD_ with the clinical gold standard method CO_PAC_ under conditions of substantial AI and further took the interplay between cardiac preload and AI into account. For this purpose we introduced a novel porcine model.

The main and most important finding of our study is that CO_TCPTD_ and CO_PAC_ were interchangeable in substantial AI. We were able to demonstrate that despite substantial AI, CO_TCPTD_ still reliably measured CO with a good ability to trend CO-changes induced by fluid loading or unloading in our porcine model.

For some decades, CO-monitoring has been used to guide hemodynamic therapy at the bedside. Since it has been suspected–and has never been disproved- that TCPTD might be relevantly confounded by AI, the less-invasive TCPTD approach has been withheld in a lot of clinical situations in patients with known or suspected AI. However, CO method comparison studies have rarely been performed under conditions of AI. Thus, our knowledge of whether hemodynamic monitoring devices provide reliable measurements in AI was very limited [8, 14, 25, 26]. Two factors contribute to this knowledge gap:

1. In clinical settings conditions often are too dynamic and varied to gather reproducible high-quality data of direct comparative assessments between AI and competent aortic valve function. Although before and after aortic valve surgery or balloon aortic valvuloplasty hemodynamic measurements in conditions with and without AI can directly be compared, in these clinical settings important confounding factors (e.g. impact of extracorporeal circulation, hypothermia, cardioplegia, thoracotomy, residual regurgitation, additional valve dysfunctions and hemodynamic disturbances after valvuloplasty [9]) must be taken into account.
2. Data from large animal trials is lacking. Earlier established techniques for experimental induction of AI were most commonly based on irreversible destruction of the aortic valve cups [27–29]. In 1930 Wiggers et al. [30] introduced a reversible technique by spreading the aortic valve cups using a transmyocardial approach. In 1970 Spring et al. [31] introduced a transvascular approach for this “aortic-valve spreading technique”, but fatal outcomes due to damage to the aorta have been reported [32]. We present a novel simple percutaneous, transcatheter approach with a self-expanding Dormia basket to spread the aortic valve in a porcine model (Fig 1). This novel AI model is innovative and promising for further hemodynamic method comparison studies, since serial measurements with varying degrees of AI can be performed, while the integrity of the myocardium and the aortic valve cups is preserved. Serial, reproducible, temporary induction of AI was an important prerequisite for comparative hemodynamic assessments during varying combinations of aortic valve and cardiac preload conditions in our study.

Reliability and trending capability of thermodilution CO in AI is poorly described [8, 14]. In 1986 Hillis and co-authors found a relevant percentage difference between the transcardiopulmonary indocyanine green dye dilution technique and the Fick method in 7 patients with left-sided valvular regurgitation who underwent cardiac catheterization (4 patients with AI and 3 with mitral valve insufficiency) [33]. Breukers et al. [14] found a significant correlation between CO_TCPTD_ and CO_PAC_ in a cohort of 8 patients with residual left-sided valvular insufficiencies after valvular surgery (minimal AI in 4 and minimal to moderate mitral valve insufficiency in 6 patients). However, fluid-induced changes of CO_TCPTD_ and CO_PAC_ did not correlate [14]. A previous clinical study by our group [8] in 18 patients undergoing transcatheter aortic valve implantation found acceptable agreement between stroke volume measured by transcardiopulmonary thermodilution and transesophageal echocardiography in patients with AI after balloon aortic valvuloplasty. However, comparative assessments with CO_PAC_ were not provided, systematic fluid challenges were absent and thus the impact of preload changes could not be taken into account. The study was primarily designed to investigate the reliability of hemodynamic monitoring in aortic valve stenosis [8].

The present study found that transcardiopulmonary thermodilution reliably measured CO, even in the most unfavorable combination of substantial AI and severe hypovolemia with a good ability to trend CO-changes induced by cardiac preload changes in comparison with the clinical gold standard method CO_PAC_ in a porcine model. Thus in everyday clinical practice TCPTD represents a less invasive alternative to PAC to measure CO in individual with known or suspected AI in the intensive care unit or operating theater.

We found a small bias between CO_TCPTD_ and CO_PAC_. This finding is consistent with those from previous clinical [34] and animal trials [13]. CO_TCPTD_ was most often found to be higher than the corresponding CO_PAC_ [13, 34, 35].

Furthermore, we found slightly higher PE values in conditions with low cardiac preload and, to a smaller extent, in conditions with substantial AI. However, these findings have to be interpreted with care and do not argue for a clinically relevant effect of thermal indicator loss promoted by aortic regurgitation on CO_TCPTD_. In fact, it should be remembered that agreement between CO_TCPTD_ and CO_PAC_ might be affected by low CO states [8, 12, 13, 34, 36]. Since CO significantly decreased in AI and in hypovolemia in our study, higher PE values have to be expected under these hemodynamic conditions.

This study used a porcine model. Both thermodilution techniques are designated for human use and not for a porcine model. Although pigs are known to have comparable hemodynamics to humans, caution should be exercised if our findings are generalized and extrapolated to clinical conditions in humans. All animals in our model received general anesthesia and mechanical ventilation. This may limit the applicability of our findings to awake spontaneously breathing subjects. In 16% of the cases, echocardiography revealed only mild AI with a regurgitation fraction below 30%. Our results might have been different if we were able to achieve moderate to severe AI in all cases. Our data were assessed during acute AI. In patients with chronic AI the left ventricle adapts to chronical volume load with consecutive changes of the ventricle size and function which could possibly impact CO measurement. Thus caution should be exercised if our findings are extrapolated to patients with chronic AI.

## Conclusions

Despite substantial AI, transcardiopulmonary thermodilution reliably measured CO under various cardiac preload conditions with a good ability to trend CO changes induced by preload changes in direct comparison with CO_PAC_ in a porcine model. CO_TCPTD_ and CO_PAC_ were interchangeable in substantial AI.

## References

[1] Aya HD, Cecconi M, Hamilton M, Rhodes A. Goal-directed therapy in cardiac surgery: a systematic review and meta-analysis. Br J Anaesth. 2013; 110(4): 510–517. doi: 10.1093/bja/aet020 23447502 10.1093/bja/aet020

[2] Goepfert MS, Richter HP, Zu Eulenburg C, Gruetzmacher J, Rafflenbeul E, Roeher K, et al Individually optimized hemodynamic therapy reduces complications and length of stay in the intensive care unit: a prospective, randomized controlled trial. Anesthesiology. 2013; 119(4): 824–836. doi: 10.1097/ALN.0b013e31829bd770 23732173 10.1097/ALN.0b013e31829bd770

[3] Pearse RM, Harrison DA, MacDonald N, Gillies MA, Blunt M, Ackland G, et al Effect of a perioperative, cardiac output-guided hemodynamic therapy algorithm on outcomes following major gastrointestinal surgery: a randomized clinical trial and systematic review. JAMA. 2014; 311(21): 2181–2190. doi: 10.1001/jama.2014.5305 24842135 10.1001/jama.2014.5305

[4] Nishimura RA, Otto CM, Bonow RO, Carabello BA, Erwin JP 3rd, Fleisher LA et al 2017 AHA/ACC Focused Update of the 2014 AHA/ACC Guideline for the Management of Patients With Valvular Heart Disease: A Report of the American College of Cardiology/American Heart Association Task Force on Clinical Practice Guidelines. Circulation. 2017; doi: 10.1161/CIR.0000000000000503 28298458 10.1161/CIR.0000000000000503

[5] Vahanian A, Alfieri O, Andreotti F, Antunes MJ, Baron-Esquivias G, Baumgartner H, et al Guidelines on the management of valvular heart disease (version 2012). Eur Heart J. 2012; 33(19): 2451–2496. doi: 10.1093/eurheartj/ehs109 22922415 10.1093/eurheartj/ehs109

[6] Hoiseth LO, Hoff IE, Hagen OA, Landsverk SA, Kirkeboen KA. Agreement between stroke volume measured by oesophageal Doppler and uncalibrated pulse contour analysis during fluid loads in severe aortic stenosis. J Clin Monit Comput. 2015; 29(4): 435–441. doi: 10.1007/s10877-015-9666-y 25638514 10.1007/s10877-015-9666-y

[7] Petzoldt M, Reuter DA. Cardiac output monitoring in severe aortic stenosis: Which technologies are reliable? J Clin Monit Comput. 2015; 29(4): 429–430. doi: 10.1007/s10877-015-9688-5 25808455 10.1007/s10877-015-9688-5

[8] Petzoldt M, Riedel C, Braeunig J, Haas S, Goepfert MS, Treede H, et al Stroke volume determination using transcardiopulmonary thermodilution and arterial pulse contour analysis in severe aortic valve disease. Intensive Care Med. 2013; 39(4): 601–611. doi: 10.1007/s00134-012-2786-7 23287875 10.1007/s00134-012-2786-7

[9] Petzoldt M, Riedel C, Braeunig J, Haas S, Goepfert MS, Treede H, et al Dynamic device properties of pulse contour cardiac output during transcatheter aortic valve implantation. J Clin Monit Comput. 2015; 29(3): 323–331. doi: 10.1007/s10877-014-9630-2 25355556 10.1007/s10877-014-9630-2

[10] Staier K, Wilhelm M, Wiesenack C, Thoma M, Keyl C. Pulmonary artery vs. transpulmonary thermodilution for the assessment of cardiac output in mitral regurgitation: a prospective observational study. Eur J Anaesthesiol. 2012; 29(9): 431–437. doi: 10.1097/EJA.0b013e3283542222 22569023 10.1097/EJA.0b013e3283542222

[11] Funcke S, Sander M, Goepfert MS, Groesdonk H, Heringlake M, et al Practice of hemodynamic monitoring and management in German, Austrian, and Swiss intensive care units: the multicenter cross-sectional ICU-CardioMan Study. Ann Intensive Care. 2016; 6: 49 doi: 10.1186/s13613-016-0148-2 27246463 10.1186/s13613-016-0148-2 PMC4887453

[12] Reuter DA, Huang C, Edrich T, Shernan SK, Eltzschig HK. Cardiac output monitoring using indicator-dilution techniques: basics, limits, and perspectives. Anesth Analg. 2010; 110(3): 799–811. doi: 10.1213/ANE.0b013e3181cc885a 20185659 10.1213/ANE.0b013e3181cc885a

[13] Huter L, Schwarzkopf KR, Preussler NP, Schubert H, Schreiber T. The level of cardiac output affects the relationship and agreement between pulmonary artery and transpulmonary aortic thermodilution measurements in an animal model. J Cardiothorac Vasc Anesth. 2007; 21(5): 659–663. doi: 10.1053/j.jvca.2007.01.005 17905270 10.1053/j.jvca.2007.01.005

[14] Breukers RM, Groeneveld AB, de Wilde RB, Jansen JR. Transpulmonary versus continuous thermodilution cardiac output after valvular and coronary artery surgery. Interact Cardiovasc Thorac Surg. 2009; 9(1): 4–8. doi: 10.1510/icvts.2009.204545 19383638 10.1510/icvts.2009.204545

[15] Cigarroa RG, Lange RA, Williams RH, Bedotto JB, Hillis LD. Underestimation of cardiac output by thermodilution in patients with tricuspid regurgitation. Am J Med. 1989; 86(4): 417–420. 2648822 10.1016/0002-9343(89)90339-2

[16] Balik M, Pachl J, Hendl J. Effect of the degree of tricuspid regurgitation on cardiac output measurements by thermodilution. Intensive Care Med. 2002; 28(8): 1117–1121. doi: 10.1007/s00134-002-1352-0 12185434 10.1007/s00134-002-1352-0

[17] Kilkenny C, Browne WJ, Cuthill IC, Emerson M, Altman DG. Improving bioscience research reporting: the ARRIVE guidelines for reporting animal research. PLoS Biol. 2010; 8(6): e1000412 doi: 10.1371/journal.pbio.1000412 20613859 10.1371/journal.pbio.1000412 PMC2893951

[18] Maisch S, Bohm SH, Sola J, Goepfert MS, Kubitz JC, Richter HP, et al Heart-lung interactions measured by electrical impedance tomography. Crit Care Med. 2011; 39(9): 2173–2176. doi: 10.1097/CCM.0b013e3182227e65 21666450 10.1097/CCM.0b013e3182227e65

[19] Bland JM, Altman DG. Agreement between methods of measurement with multiple observations per individual. J Biopharm Stat. 2007; 17: 571–582. doi: 10.1080/10543400701329422 17613642 10.1080/10543400701329422

[20] Critchley LA, Critchley JA. A meta-analysis of studies using bias and precision statistics to compare cardiac output measurement techniques. J Clin Monit Comput. 1999; 15(2): 85–91. 12578081 10.1023/a:1009982611386

[21] Critchley LA, Lee A, Ho AM. A critical review of the ability of continuous cardiac output monitors to measure trends in cardiac output. Anesth Analg. 2010; 111(5): 1180–1192. doi: 10.1213/ANE.0b013e3181f08a5b 20736431 10.1213/ANE.0b013e3181f08a5b

[22] Critchley LA, Yang XX, Lee A. Assessment of trending ability of cardiac output monitors by polar plot methodology. J Cardiothorac Vasc Anesth. 2011; 25(3): 536–546. doi: 10.1053/j.jvca.2011.01.003 21419654 10.1053/j.jvca.2011.01.003

[23] Saugel B, Grothe O, Wagner JY. Tracking Changes in Cardiac Output: Statistical Considerations on the 4-Quadrant Plot and the Polar Plot Methodology. Anesth Analg. 2015; 121(2): 514–524. doi: 10.1213/ANE.0000000000000725 26039419 10.1213/ANE.0000000000000725

[24] R Core Team. R: A language and environment for statistical computing R Foundation for Statistical Computing, Vienna, Austria URL https://www.r-project.org. Accessed 2017.

[25] Lorsomradee S, Cromheecke S, De Hert SG. Uncalibrated arterial pulse contour analysis versus continuous thermodilution technique: effects of alterations in arterial waveform. J Cardiothorac Vasc Anesth. 2007; 21(5): 636–643. doi: 10.1053/j.jvca.2007.02.003 17905266 10.1053/j.jvca.2007.02.003

[26] Furukawa H, Ohkado A, Nagashima M, Ohsawa H, Ichikawa S. Clinical evaluation of intraoperative cardiac output measurement by a new arterial pressure waveform analysis method (FloTrac/Vigileo) in open heart surgery. Kyobu Geka. 2013; 66(9): 775–783. 23917227

[27] Magalhaes MA, Lipinski MJ, Minha S, Escarcega RO, Baker NC, Ota H, et al Aortic valve ChromaFlo(R): a feasibility study of aortic regurgitation and effective annular aortic area assessment in a porcine model. Cardiovasc Revasc Med. 2014; 15(3): 156–159. doi: 10.1016/j.carrev.2014.02.006 24767314 10.1016/j.carrev.2014.02.006

[28] Zong GJ, Jiang HB, Bai Y, Wu GY, Ye GM, Chen JK, et al Percutaneous valved stent implantation in the ascending aorta for the treatment of very high-risk aortic regurgitation: an animal study. J Surg Res. 2013; 185(2): 940–944. doi: 10.1016/j.jss.2013.06.059 23910885 10.1016/j.jss.2013.06.059

[29] Stugaard M, Koriyama H, Katsuki K, Masuda K, Asanuma T, Takeda Y, et al Energy loss in the left ventricle obtained by vector flow mapping as a new quantitative measure of severity of aortic regurgitation: a combined experimental and clinical study. Eur Heart J Cardiovasc Imaging. 2015; 16(7): 723–730. doi: 10.1093/ehjci/jev035 25762562 10.1093/ehjci/jev035

[30] Wiggers CJ, Theisen H, Williams HA. Further Observations on Experimental Aortic Insufficiency: II. Cinematographic Studies of Changes in Ventricular Size and in Left Ventricular Discharge. J Clin Invest. 1930; 9(2): 215–233. doi: 10.1172/JCI100300 16693929 10.1172/JCI100300 PMC435692

[31] Spring DA, Rowe GG. A device for production of aortic insufficiency in intact experimental animals. J Appl Physiol. 1970; 29(4): 538–540. 5460279 10.1152/jappl.1970.29.4.538

[32] Ardehali A, Segal J, Cheitlin MD. An improved valve-spreading catheter for producing reversible graded acute aortic regurgitation. Cardiology. 1996; 87(4): 276–278. 8793158 10.1159/000177104

[33] Hillis LD, Firth BG, Winniford MD. Comparison of thermodilution and indocyanine green dye in low cardiac output or left-sided regurgitation. Am J Cardiol. 1986; 57(13): 1201–1202. 3706179 10.1016/0002-9149(86)90704-6

[34] Böck J, Barker B, Mackersie R, Tranbaugh R, Lewis F. Cardiac Output Measurement Using Femoral Artery Thermodilution in Patients. J Crit Care. 1989; 4: 106–111.

[35] Sakka SG, Kozieras J, Thuemer O, van Hout N. Measurement of cardiac output: a comparison between transpulmonary thermodilution and uncalibrated pulse contour analysis. Br J Anaesth. 2007; 99(3): 337–342. doi: 10.1093/bja/aem177 17611251 10.1093/bja/aem177

[36] Renner LE, Morton MJ, Sakuma GY. Indicator amount, temperature, and intrinsic cardiac output affect thermodilution cardiac output accuracy and reproducibility. Crit Care Med. 1993; 21(4): 586–597. 8472580 10.1097/00003246-199304000-00021

